# Metabolic risk stratification of night shift workers in a large retail workplace through clustering and SHAP interpretation

**DOI:** 10.3389/fpubh.2025.1704046

**Published:** 2026-01-12

**Authors:** InHo Lee, SangHee Hong, EunChul Jang, JuneHee Lee, JeongBeom Lee

**Affiliations:** 1 Department of Occupational and Environmental Medicine, Soonchunhyang University Cheonan Hospital, Cheonan, Republic of Korea; 2 Department of Physiology, Soonchunhyang University College of Medicine, Cheonan-si, Republic of Korea; 3 Soonchunhyang University College of Medical Sciences, Asan-si, Republic of Korea; 4 Department of Occupational and Environmental Medicine, Soonchunhyang University Hospital, Yongsan-gu, Republic of Korea

**Keywords:** night shift work, metabolic risk, clustering, explainable machine learning, occupational health

## Abstract

**Background:**

While working at night is an important occupational risk factor associated with metabolic diseases, the fact that different workers have different risk patterns has not been fully studied. In this study, we used explainable AI analysis methods to find out and specify what factors contributed to metabolic health risks among night shift workers.

**Materials and methods:**

The study was conducted with employees working at night at a large domestic distribution company. Basic information such as health examination data (blood sugar, cholesterol, blood pressure, etc.) and age and working period of the study subjects was collected and analyzed. Using unsupervised learning (UMAP and K-means clustering), people with similar health characteristics were divided into four groups. Using a prediction model called Random Forest and an analysis of Shapley Additive Explanations (SHAP), we found out what health factors had the greatest impact on distinguishing these four groups.

**Results:**

In this study, four health types (clusters) were identified. Cluster 0 is an overall healthy low-risk group, which seems to reflect the “health worker effect.” Cluster 1 included workers who were older (average 40.9 years), had longer night work experience (average 13.3 years), and had high blood pressure and cholesterol levels. Cluster 2 is a group of moderate-risk groups with slightly increased body mass index (BMI) and lipid levels, which are interpreted as transitional conditions with deteriorating health. Cluster 3 was found to be young (average 37.4 years), had a short working period (average 6.0 years), and had high BMI, blood sugar, and blood pressure, which led to many irregularities in lifestyle and social jet lag. In addition, because of SHAP analysis, BMI, triglyceride-blood sugar index, blood pressure, and working period were identified as the most important factors in distinguishing these four groups.

**Conclusion:**

It has been confirmed that the metabolic health risks of night workers vary from person to person, and that these differences can be effectively classified (tiered) through machine learning analysis. The results of this study serve as an important foundation for developing customized industrial health strategies, such as redesigning work schedules or personalized health care and prevention programs.

## Introduction

Night and shift workers now comprise an estimated 15–30% of the global workforce, based on cross-sectional labor force surveys from the ILO and other national datasets (1). Night and shift work has been found to be the major factors in metabolic and cardiovascular health deterioration. The 2024 review ‘Shift Rhythm’ concluded that shift work increases the risk of myocardial infarction, coronary artery disease, hypertension, atherosclerosis, and metabolic syndrome (2–4). Shift work increases the likelihood of oxidative damage and involvement of inflammatory biomarkers by factors such as circadian rhythm disorders, unhealthy eating habits, and mental and physical stress (3). According to the WHO/ILO Joint Estimates, long working hours are associated with a higher risk of ischemic heart disease and stroke (5).

Recent cohort research indicates that the combination of night work and extended working hours elevates cerebrovascular disease and myocardial infarction (3, 6). Night shifts have been shown to increase the risk of developing type 2 diabetes with longer exposure and increase the risk if the working period is more than 10 years (7, 8). Night work is associated with melatonin inhibition, insulin resistance, and circadian disturbance, and these alterations have been proposed as mechanisms that may contribute to increased diabetes risk (9). Such disruptions in circadian regulation and metabolic homeostasis have been discussed as possible contributors to metabolic syndrome (9). Night shift workers were more vulnerable to metabolic syndrome components than day workers (10, 11). Shift work causes impaired blood sugar control, changes in insulin secretion rhythm, delayed cortisol secretion, and cholesterol imbalance (11). The main cause of the increase in obesity due to shifts and night shifts is known as ‘night eating’ and ‘sleep debt’ (11, 12). General lifestyle factors are known to have a higher risk of obesity when combined with lack of exercise, late-night eating, drinking, or using cigarettes and night shifts (13). Therefore, the health problems associated with shift work should be understood as a complex condition ‘Circadian Syndrome’ rather than isolated issues such as shortened sleep, circadian disruption, or mental health difficulties (14).

Night and shift work is also closely related to sleep disorders, depression, anxiety, and burnout, and shift workers have lower job satisfaction, more fatigue and sleep deprivation than day workers, and can be accompanied by a variety of mental health problems (3). It is known that frequent ‘quick returns’ after less than 11 h of rest after night shift can worsen depression, anxiety and cognitive decline (15). Social lag caused by night shifts also increases stress in family and social life, leading to a vicious cycle (16) that worsens health behavior. From a policy and institutional point of view, the European Union Working Hours Directive specifies a continuous 11-h daily break and a minimum 24-h weekly break (and 11-h daily break) per 7 days as minimum criteria, and the average night shift is not to exceed 8 h per 24-h period (17).

This regulation is based on the rationale that long working hours, a study by WHO and ILO, causes stress and disease (1). In South Korea, the Korea Working Conditions Survey (KWCS) has comprehensively captured data on irregular and night work frequency, daytime work hours, work intensity, sleep, and mental health. The KWCS data reported associations between irregular work and musculoskeletal pain, headache or eye fatigue, reduced well-being, and increased depression (18–20). South Korea’s working hours are longer than the average of the Organization for Economic Cooperation and Development (OECD), ranking fourth in the OECD (21). There is a 52-h workweek, but unlike the EU, there is no separate upper limit for night shifts, creating an institutional void (22). Recent government discussions on working hours flexibility have raised concerns among experts that working hours irregularities and the risk of short-term overwork (23) may increase.

As previously noted, most previous studies have evaluated single outcomes based on sleep quality or metabolic indicators or focused on single risk factors and interventions (6, 9, 10). Although international organizations have established guidelines for regulating working hours and mitigating the health effects of long working hours (1, 5), there is a lack of evidence for integrated management of multidimensional factors at the workplace level (3). Therefore, such field integrated studies are still rare, and this study seeks to bridge this gap. This study aims to comprehensively assess physiological, metabolic, cardiovascular, and sleep health indicators in night workers at large retail establishments, and to group workers to explain the need for integrated management. It also aims to provide evidence-based recommendations for the health care of shift workers.

## Materials and methods

### Study population

The study was conducted based on the health examination index of night workers at a large distribution site located in Cheonan, South Chungcheong Province, and 320 employees worked at this site in 2024. Among them, 28 employees handling other harmful substances under the Korea Occupational Safety and Health Act were excluded from the study, and 73 people already under management due to hypertension, diabetes, and dyslipidemia were also excluded, so the final 219 people were selected as subjects for integrated management. According to the current Republic of Korea Occupational Safety and Health Act, night workers are defined as people (24) who work more than four times a month for 8 h for more than 6 months from 12 a.m. to 5 p.m., or work an average of 60 h a month from 10 p.m. to 6 p.m. Due to the nature of the workplace, the night shift schedule was regular rotational, the employees did not work in isolation, and the night shift was in compliance with the break time prescribed by law in Korea.

### Characteristics of the workplace

This study was conducted on large warehouse-type distribution businesses, and the facilities consist of various departments such as food courts, fresh food (wholesale, bakery, deli, seafood), product display and logistics, check-out and customer service, parking and cart management, and marketing. Looking at the characteristics of each department, first of all, employees of food-related departments generally work in a refrigerated or frozen environment and are responsible for shelf-life management and waste disposal. In addition, the actual working hours are often extended as the furloughed workers often have to perform inventory collection, processing, and cleaning tasks. The product display and logistics departments work mainly at dawn and have a high-intensity manual labor to repeatedly transport and organize heavy goods. The check-out and customer service departments have a high proportion of emotional labor as they handle direct cash transactions and customer complaints. In addition, the parking and cart collection departments may increase outdoor work or increase labor intensity depending on the season. The workplace complies with the Korean Labor Standards Act of 52 h a week, and the operating hours are from 10 a.m. to 10 p.m. Logistics, cooking, and cleaning tasks performed before and after business hours are the main causes of night and long working hours.

### Data collection

For the health screening data analysis, records were used that included demographic information (age), night shift work period (shift_years), anthropometric measurements (BMI), blood pressure (systolic [SBP] and diastolic [DBP]), fasting blood tests (glucose), lipid panels (total cholesterol, triglycerides, high-density lipoprotein [HDL] cholesterol, and low-density lipoprotein [LDL] cholesterol), and insomnia scores.

Height and body weight were measured using an automated stadiometer (InBody 520; Biospace Inc., Seoul, Korea) with participants wearing light clothing and no shoes. BMI was calculated as weight (kg) divided by height squared (m^2^) (25). Systolic and diastolic blood pressures were measured twice in a seated position after 5 min of rest using an automated sphygmomanometer (Omron HEM-7080IT, Kyoto, Japan); the mean of the two readings was recorded. Blood samples were collected after an overnight fast of at least 8 h and analyzed in a certified clinical laboratory following standard enzymatic procedures. Insomnia symptoms were assessed using the Insomnia Severity Index (ISI), a 7-item self-report questionnaire rated on a 5-point Likert scale (0–4). The total score ranges from 0 to 28, with higher scores indicating more severe insomnia. The ISI has demonstrated excellent internal consistency (Cronbach’s *α* = 0.90) and strong convergent validity with objective sleep parameters (26).

The number of years at night work was also considered a key factor in this study. All variables were scaled using standardization (z-scores).

### Data analysis

For data analysis in this study, Python 3.13 was used. For unsupervised learning, scikit-learn’s K-means was applied as the primary algorithm. Uniform Manifold Approximation and Projection (UMAP) from umap-learn was utilized for understanding the structure and visualizing high-dimensional variables. A predictive model was built using RandomForestClassifier, and variable contributions were evaluated using a combination of Gini-based internal importance and permutation importance. To enhance interpretability, global and local Shapley Additive Explanations (SHAP) values were calculated using the shap package.

### Unsupervised clustering

To explore health risk profiles, we used a two-step procedure. First, UMAP was applied to standardized data (Z-score transformation) to reduce dimensions while preserving local structures. Second, we performed K-means clustering on 2D UMAP embeddings and applied the commonly used settings n_neighbors = 15, min_dist = 0.1, and random_state = 42 (27–29). This combination of parameters is known to be effective in reliably preserving inter-cluster structures and in deriving reproducible results, which have been conventionally adopted in several prior studies.

The same parameter settings were maintained in all analyses to ensure consistency and reproducibility of the analyses. Whether the cluster-specific analysis reflected the differentiation of the actual clinically significant health risk groups (e.g., blood pressure, BMI, triglycerides, etc.) and evaluated mathematically good analysis.

### Supervised modelling and interpretation

To investigate the variables that strongly distinguished the clusters, a Random Forest classifier was trained to predict cluster membership using the original standardized variables as input. In this study, considering that the total number of samples is limited to 200, model performance and feature-importance stability were evaluated using a repeated 5-fold cross-validation scheme (10 repetitions). A Random Forest classifier (n_estimators = 500, class_weight = “balanced”) was employed. Cross-validated accuracies, precisions, recalls, and F1-scores were computed for each repetition and summarised in terms of sample mean, standard deviation and range (30, 31).

To interpret the relative importance of the variables that contributed to the prediction, the SHAP value was calculated, and a SHAP summary plot was created for each cluster to visualize the contribution distribution of the main variables. This allowed us to identify the key variables that contributed to distinguishing each cluster.

## Results

### Overall characteristics of study participants

A total of 219 participants were included in the final analysis. The mean age was 38.7 years, and 44% were female. Average BMI was 24.1, and the mean shift work tenure was 8.3 years. Full descriptive statistics are presented in Table 1.

**Table 1:** Physical characteristics of subjects (*n* = 219).

Statistic	Age	BMI	SBP	DBP	FBS	Chol total	TG	HDL	LDL	ISI
Mean	38.53	24.91	128.28	80.00	98.12	207.43	132.35	60.58	120.00	5.76
Std	6.88	4.21	11.08	7.97	18.19	37.22	114.85	17.56	33.04	4.39
Min	24	16.9	100	59	57	121	34	27	14	0
25%	34	22.1	121	75	91	182	70.5	48	98.5	2
50%	38	24.4	130	80	96	203	99	57	118	5
75%	43	27.3	134	85	102	229	152	71	142.5	9
Max	58	42.7	185	110	258	351	1,046	141	236	20

BMI, body mass index; SBP, Systolic Blood Pressure; DBP, Diastolic blood pressure; FBS, Fasting Blood Sugar; Chol, Cholesterol; TG, Triglyceride; HDL, High-Density Lipoprotein; LDL, Low-Density Lipoprotein; ISI, Insomnia Severity Index.

### Silhouette score and cluster determination

**Figure 1:**
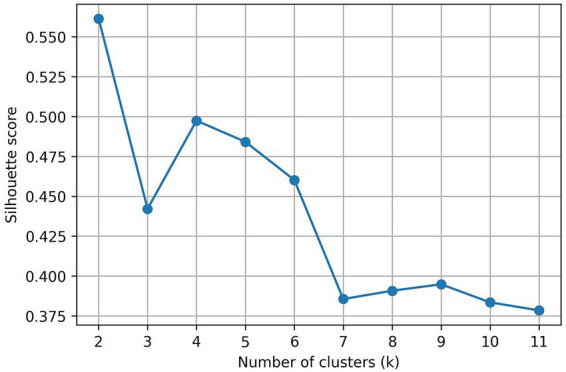
Silhouette scores for different *k*. To verify the feasibility of clustering, the silhouette coefficient was calculated by varying the number of clusters from 2 to 11. As a result, the score (0.5614) was the highest when *k* = 2, showing the most distinctive structure when divided into two groups. However, considering the breadth of interpretation and the purpose of the study, the analysis was conducted further by increasing the number of clusters. Among them, *k* = 4 (0.4974) has relatively high scores, rather than simply dividing into two groups, it was judged to be a structure that could reflect various health characteristics. On the other hand, from *k* = 7 or higher, the score fell below 0.40, and the distinction between clusters tended to become unclear. Taking together, clustering in sections *k* = 3 to 5 is not completely optimal, it provides meaningful classification results in terms of interpretation and practical application.

Using the number of clusters (k) from 2 to 11 silhouette scores as an evaluation indicator by applying the K-means algorithm (Figure 1), the silhouette coefficient at k = 2 was the highest at 0.5614, but we looked at the case of k = 4 with the next highest silhouette coefficient of 0.4974. For K = 4, it was the best fit for the validity of the cluster structure as it was close to 0.5 with k = 2. This study ultimately set both k = 2 and k = 4 clusters as analysis units, and subsequent cluster characteristic analysis and predictive modeling were conducted.

### K-means clustering with UMAP visualization

**Figure 2:**
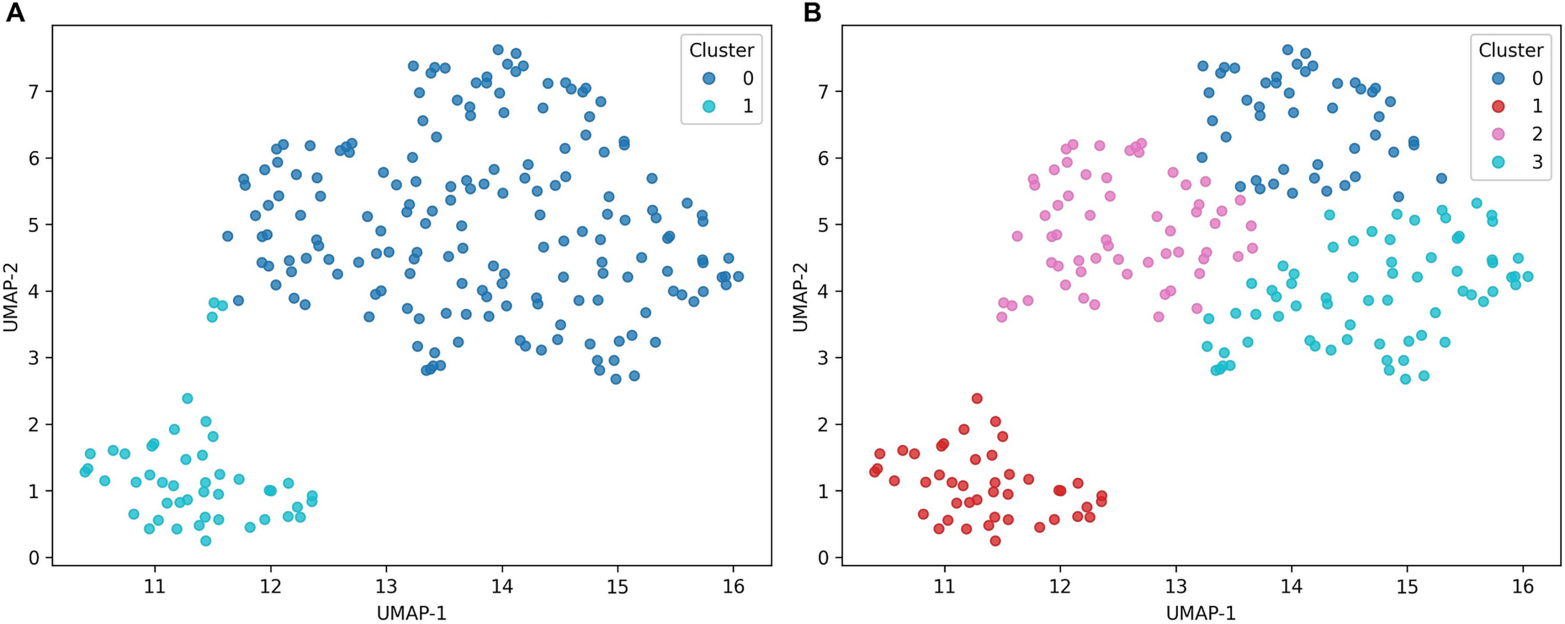
UMAP visualization of *K*-means clustering results. **(A)** *K* = 2, all workers are classified into two simple clusters, showing a reduced detailed heterogeneity. **(B)** *K* = 4, four clusters are more clearly divided, showing distinct characteristics according to age, shift work period, and metabolic index combination.

Figure 2 shows the clustering results for k = 2 and k = 4. For k = 2, the cluster was visually well separated with minimal overlap along both embedding axes (Figure 2A). For k = 4, we classified the entire dataset into four distinct subgroups (Figure 2B). At k = 4, the cluster showed distinguishable patterns in key features such as shift year, age, and metabolic profile (e.g., blood pressure, cholesterol), demonstrating the interpretability of subgroups by more clinical factors. These results suggest that the multi-group classification can more accurately reflect the physiological and working time factors of the subjects than simple binary segmentation.

### Cluster characteristics (k = 2)

**Figure 3:**
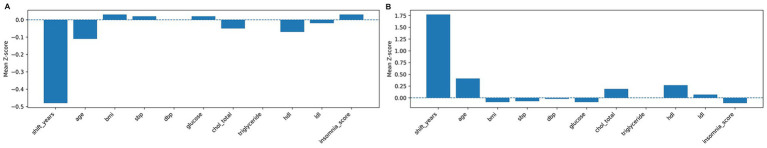
Mean *Z*-scores of clusters identified with *k* = 2. **(A)** Cluster 0 shows lower values for shift years and age, representing younger workers with shorter shift experience. **(B)** Cluster 1 shows higher values for shift years and age, representing long-term shift workers with older age. S, shift_years, night shift work period; BMI, body mass index; SBP, systolic blood pressure; DBP, diastolic blood pressure; glucose, fasting blood sugar; chol_total, total cholesterol; HDL, high-density lipoprotein; LDL, low-density lipoprotein.

For the two clusters, a comparison of the mean Z-scores of the main variables first showed that cluster 0 showed a markedly lower negative value in the shift work year (Z ≈ −0.48, average = 5.83 years) and was below average age (Figure 3A). This could be seen as a young, short-term shift work group, and other metabolic and sleep-related indicators did not show any significant deviation from the mean. Cluster 1 showed a markedly high positive value in the shift work year (Z ≈ +1.75, average = 12.5 years), and the age was also relatively high (Figure 3B). This cluster could be interpreted as an older, longer-term shift group, with total cholesterol, HDL, and LDL above average, while triglycerides remained around the population mean. Conversely, BMI, blood pressure (SBP and DBP), fasting blood glucose, triglyceride, and insomnia scores were generally below average or showed no significant difference (Figure 3A). In summary, the k = 2 cluster was largely separated by the difference between shift work period and age, which exhibited distinct characteristics between the long-term and early-shift groups.

### Cluster characteristics (k = 4)

**Figure 4:**
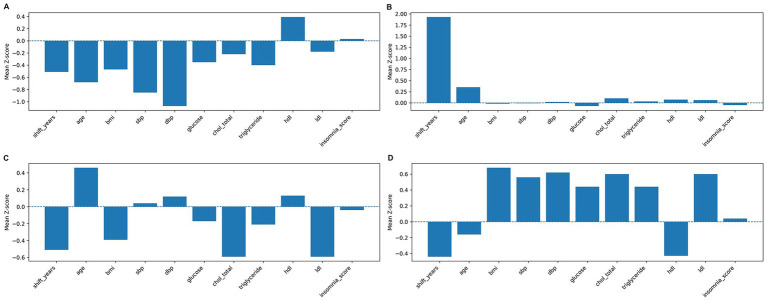
Mean *Z*-scores of clusters identified with *k* = 4. **(A)** Cluster 0: low-risk group with overall below-average metabolic indicators. **(B)** Cluster 1: long-term shift workers with older age. **(C)** Cluster 2: intermediate group with moderate age and mixed metabolic profiles. **(D)** Cluster 3: younger workers with elevated metabolic risk (BMI, blood pressure, glucose, lipids). S, shift_years, night shift work period; BMI, body mass index; SBP, systolic blood pressure; DBP, diastolic blood pressure; glucose, fasting blood sugar; chol_total, total cholesterol; HDL, high-density lipoprotein; LDL, low-density lipoprotein.

For the four clusters, to find out about the mean Z-scores of the key variables, Cluster 0 showed below-average values for shift work tenure, age, and most metabolic indicators, with particularly low values for blood pressure and blood glucose (Figure 4A). This can be viewed as a low-risk group or a group with relatively good health indicators. Cluster 1 showed distinct positive values for shift work tenure (Z ≈ +2.0, Mean = 13.3 years) and age (Z ≈ +0.3, Mean = 40.9 years) with no significant deviation in other metabolic indicators. This cluster is interpreted as an older group with long-term shift work experience (Figure 4B). Cluster 2 had a slightly higher age and showed an increase in some lipid indicators but had low shift work tenure. Additionally, total cholesterol and LDL were below average. This can be interpreted as a group with a moderate level of metabolic risk and age distribution (Figure 4C). Cluster 3 had low shift work tenure and age, but showed above-average values for BMI, blood pressure (SBP, DBP), blood glucose, and lipids (total cholesterol, triglycerides, and LDL). This cluster can be characterized as a young shift work group with high metabolic risk (Figure 4D).

In summary, the k = 4 clusters were categorized as (1) a low-risk, healthy group, (2) a long-term shift work, relatively older group, (3) a moderate-risk group, and (4) a relatively young shift work group with high metabolic risk. This shows that employee groups can be distinctly characterized by a combination of shift work tenure, age, and metabolic indicators. Accordingly, the results of the supervised modeling and interpretation will be explained below, based on the k = 4 solution.

### Cross-validated random Forest classification performance

Cross-validated evaluation of the Random Forest classifier showed that both clustering solutions (k = 2 and k = 4) achieved stable and reproducible performance. For the k = 2 model, the mean accuracy across 10 × 5 repeated cross-validation was 0.976 ± 0.0027 (range 0.968–0.977), and macro-averaged precision, recall, and F1-scores were likewise consistent (0.971 ± 0.0017, 0.960 ± 0.0069, and 0.964 ± 0.0045, respectively). For the k = 4 solution, the classifier also demonstrated robust performance, with an accuracy of 0.897 ± 0.016 (range 0.872–0.918) and stable macro-precision, recall, and F1-scores (0.904 ± 0.014, 0.901 ± 0.016, and 0.899 ± 0.016, respectively).

Cluster-specific performance metrics for both k = 2 and k = 4 including mean ± SD and the observed minimum–maximum ranges are summarized in Table 2. Overall, the model consistently reproduced the underlying cluster structure across repeated resampling, indicating strong classification stability and supporting the interpretability of the derived subgroup patterns.

**Table 2:** Cluster-specific cross-validated classification performance (10 repetitions × 5 folds).

k	Cluster	Metric	Mean ± SD	Range (Min–Max)
2	0	Precision	0.982 ± 0.021	0.944–1.000
		Recall	0.988 ± 0.017	0.941–1.000
		F1-score	0.985 ± 0.013	0.955–1.000
	1	Precision	0.961 ± 0.055	0.818–1.000
		Recall	0.932 ± 0.079	0.778–1.000
		F1-score	0.944 ± 0.050	0.857–1.000
4	0	Precision	0.869 ± 0.103	0.667–1.000
		Recall	0.816 ± 0.122	0.444–1.000
		F1-score	0.833 ± 0.083	0.615–1.000
	1	Precision	0.959 ± 0.053	0.818–1.000
		Recall	1.000 ± 0.000	1.000–1.000
		F1-score	0.979 ± 0.028	0.900–1.000
	2	Precision	0.826 ± 0.089	0.625–1.000
		Recall	0.848 ± 0.114	0.417–1.000
		F1-score	0.830 ± 0.076	0.556–1.000
	3	Precision	0.917 ± 0.073	0.765–1.000
		Recall	0.880 ± 0.069	0.714–1.000
		F1-score	0.895 ± 0.047	0.800–1.000

Values are reported as mean ± standard deviation (SD) and the observed range Minimum (Min)–Maximum (Max) across repetitions.

### SHAP analysis by cluster

**Figure 5:**
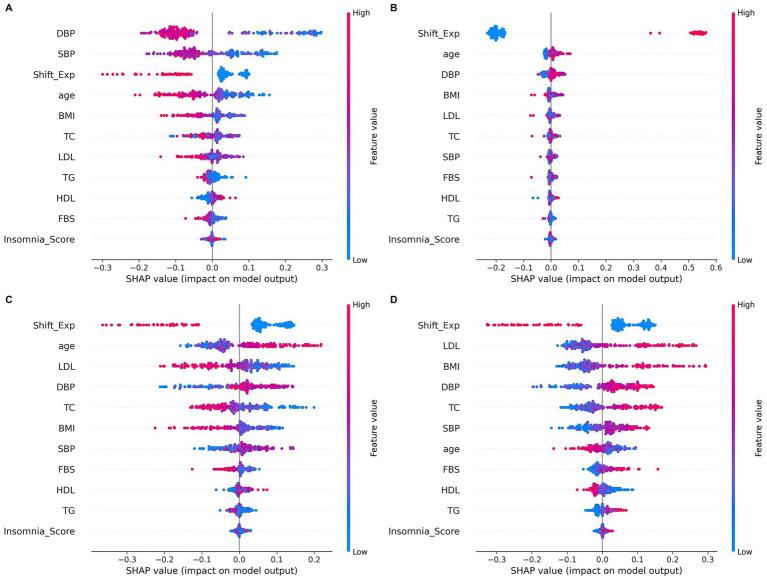
SHAP summary plots for four clusters. **(A)** Cluster 0: Systolic blood pressure and diastolic blood pressure had the greatest impact, and age and shift work experience were also important factors. **(B)** Cluster 1: The impact of shift_years is overwhelming, showing that long-term work is a major factor in determining cluster characteristics. **(C)** Cluster 2: Metabolic indicators such as LDL, total cholesterol, and BMI have a significant impact and are combined with shift work experience to separate clusters. **(D)** Cluster 3: LDL, BMI, and blood pressure indicators were important factors, especially those related to lipid metabolism. S, shift_years, night shift work period; BMI, body mass index; SBP, systolic blood pressure; DBP, diastolic blood pressure; glucose, fasting blood sugar; chol_total, total cholesterol; HDL, high-density lipoprotein; LDL, low-density lipoprotein.

A SHAP analysis was used to identify the variables contributing to the classification of each cluster. Overall, shift work experience (Shift_Exp) emerged as a consistently important variable across all clusters, showing that the type and duration of work are key factors in cluster differentiation. In Cluster 0, SBP and DBP had the greatest impact on the model’s output, with age and shift work experience also playing significant roles (Figure 5A). Cluster 1 showed an overwhelmingly large contribution from shift work experience, confirming that long-term tenure is a major factor in determining cluster characteristics (Figure 5B). In Cluster 2, metabolic indicators such as LDL, total cholesterol (TC), and BMI had a relatively large influence, combining with shift work experience to distinguish the cluster (Figure 5C). Finally, in Cluster 3, LDL, BMI, and blood pressure indicators were important factors, with a particularly high contribution from lipid metabolism-related indicators (Figure 5D).

As such, the SHAP analysis shows that while each cluster is commonly influenced by shift work tenure, they are also specifically differentiated by a variety of physiological factors such as blood pressure, lipids, BMI, and age.

## Discussion

This study combined UMAP-K-means clustering and random forest-SHAP interpretation for shift workers to show that metabolic risk characteristics can be structured into heterogeneous subgroups. In the search for the optimal number of clusters, the silhouette coefficient was highest at k = 2 (0.5614), but the cluster solution oversimplified the participant structure by merging different subgroups metabolically. Investigating the multigroup interpretation of k = 3 to 5, which considered interpretability and subjects of public health intervention, revealed the most pronounced profile of k = 4 (0.4974) according to the combination of age, duration of shift, blood pressure, lipid, BMI, and blood glucose. Each cluster showed a distinct combination of shift period, age, metabolic indicators (low-risk group, long-term exposure, moderate-risk group, and young high-risk group). From a public health perspective, these four clusters were able to distinguish subgroups that required distinct prevention or management strategies, allowing them to choose them as the best model for future interpretations (32).

This study is a case of applying a machine learning approach in a single workplace to identify heterogeneous metabolic risk groups based on a combination of night shift experience and physiological indicators. Workers were divided into several subgroups according to the patterns of shift duration, blood pressure, lipid, BMI, and blood sugar, and random forest-SHAP analysis confirmed that night shift experience was consistently the most important contributing factor in each cluster. Notably, a “young high-risk group” (cluster 3, Figure 4D) with a short shift work period but a concentration of metabolic risk factors such as BMI, blood pressure, and blood sugar was identified. These structures suggest risk heterogeneity within the shift worker population and highlight the need for customized interventions (33). These findings are closely related to the accumulation of metabolic risk over time in addition to the duration of shift exposure, suggesting that risk heterogeneity is evident even within the same shift worker group (34). For example, cluster 0 (Figures 4A, 5A) was a low-risk group, showing below-average or good health status across all indicators. In fact, some studies have reported that stress responses or metabolic abnormalities may not be noticeable during the early stages of shift work (35). However, these protective effects are likely temporary and may increase the risk at long-term follow-up (36, 37). Meanwhile, cluster 1 (Figures 4B, 5B) was the group with high shift period and age, which showed high blood pressure and high lipid levels. This is interpreted to be due to the accumulation of night exposure and worsening of metabolic risk with age.

Previous studies (38–40) reported that long night shifts increased the risk of metabolic syndrome, type 2 diabetes, and cardiovascular disease, which supports the interpretation of results in the long-term exposed and older population groups identified in this study. The random forest model demonstrated stable performance, as evidenced by the repeated 10 × 5 cross-validation results (mean accuracy 88.72%, SD 4.55%). Clusters 1 and 3 showed high precision and reproducibility, which distinguished distinct physiological differences between the long-term working group and the young high-risk group. On the other hand, some overlap was observed in clusters 0 and 2, which can be interpreted as a metastatic characteristic indicating an intermediate risk stage or an early state of adaptation. As a result of SHAP analysis, metabolic indicators such as shift work period, blood pressure, BMI, and lipids were identified as the most influential factors (41), and this RF-SHAP analysis procedure has significance in that it not only distinguishes cluster structure predictively but also enhances semantic interpretation in terms of actual occupational health.

### Limitations

Although this study looked at metabolic health risks in shift workers through cluster analysis and SHAP interpretation, it has some limitations. First, since it is a cross-sectional study design, it is difficult to directly determine the causal relationship between shift work and metabolic abnormalities. Second, there is a limit to generalizing results for other industries or occupations because it was only targeted at workers in one workplace. Third, because it included self-report questionnaire data such as work type and lifestyle, there is a possibility of bias in response. Fourth, although the number of clusters was determined by the silhouette factor, this method may not fully reflect the clinical implications of all clusters. Fifth, the number of samples is limited, so the accuracy of 87.9%, which is the result of holdout validation of the random forest model, needs to be interpreted carefully. Sixth, since only night workers were included in the study, comparative analysis with day workers or shift workers was impossible. In the future, research including various types of work is needed. Finally, due to the lack of long-term follow-up results such as metabolic syndrome or cardiovascular disease incidence, it was not possible to assess whether the health risk of each cluster led to actual disease.

## Conclusion

This study used machine learning and SHAP techniques together to systematically analyze the metabolic health risks of shift workers. As a result, the metabolic risks of shift workers were not all the same, and they could be divided into four types of groups according to the combination of age, length of service, BMI, blood pressure, blood sugar, and lipid levels. In particular, the group of older population workers who worked shift for a long period of time and the group that focused on metabolic risk factors were identified as high-risk groups, showing that priority health care and intervention were needed. In addition, the SHAP analysis clarified the key factors that distinguish each group, confirming that the results of this study can be used in practice for interpreting health examination results, early screening of high-risk groups, and improving the design of work schedules.

## Data Availability

The raw data supporting the conclusions of this article will be made available by the authors, without undue reservation.
